# Non-invasive multimodal imaging of Diabetic Retinopathy: A survey on treatment methods and Nanotheranostics

**DOI:** 10.7150/ntno.56015

**Published:** 2021-01-15

**Authors:** Rajkumar Sadasivam, Gopinath Packirisamy, Snehlata Shakya, Mayank Goswami

**Affiliations:** 1 Divyadrishti Imaging Laboratory, Department of Physics, Indian Institute of Technology Roorkee, Roorkee, Uttarakhand-247667, India.; 2 Nanobiotechnology Laboratory, Department of Biotechnology, Indian Institute of Technology Roorkee, Roorkee, Uttarakhand-247667, India.; 3 Department of clinical physiology, Lund University, Skåne University Hospital, Skåne, Sweden.

**Keywords:** diabetic retinopathy, imaging biomarkers, treatment, drugs and nano-theranostics

## Abstract

Diabetes Retinopathy (DR) is one of the most prominent microvascular complications of diabetes. It is one of the pre-eminent causes for vision impairment followed by blindness among the working-age population worldwide. The de facto cause for DR remains challenging, despite several efforts made to unveil the mechanism underlying the pathology of DR. There is quite less availability of the low cost pre-emptive theranostic imaging tools in terms of in-depth resolution, due to the multiple factors involved in the etiology of DR. This review work comprehensively explores the various reports and research works on all perspectives of diabetic retinopathy (DR), and its mechanism. It also discusses various advanced non-destructive imaging modalities, current, and future treatment approaches. Further, the application of various nanoparticle-based drug delivery strategies used for the treatment of DR are also discussed. In a nutshell, the present review work bolsters the pursuit of the development of an advanced non-invasive optical imaging modal with a nano-theranostic approach for the future diagnosis and treatment of DR and its associated ocular complications.

## Introduction

Diabetic retinopathy (DR) is a major complication of diabetes and one of the main causes of vision impairment and blindness among people worldwide. The prevalence of the DR is about one-third of the diabetes patients worldwide; in which one-tenth of the patients have vision impairment with increasing proportion 1,2. It is quite essential to study DR because retina being the part of the brain which endeavor the opportunity to study the neurodegenerative and vascular changes. Besides, DR is also associated with other complications such as cardiovascular disease and stroke 3,4, nephropathy 5,6, etc. Moreover, there is controversy over the vasculopathy or neuropathic nature of ocular diabetic complications. After extensive retinal neurodegenerative studies in animals, it is widely recognized that DR causes both neuropathy and vasculopathy. Researchers are still posing questions about whether neuropathy precedes vasculopathy or vice versa. The circumstantial evidence suggested the hypothesis that DR initiates with neuropathy and subsequently causes diabetic microvascular retinopathy 7,8. Sohn et al. 2016 confirmed that the diabetic retinal neurodegeneration is preceded by clinical diabetic retinopathy through experiments on mouse models 9. These findings advocated that DR is a neurodegenerative disorder that leads to the progressive disruption of various cell types of retina 10. The neurovascular cause of DR is associated with the blood-retinal-barrier (BRB) consisting of the interdependence of neurons, glial cells, and vasculature of the central nervous system 11,12. Also, vision impairment is closely related to glioblastoma. The worsening visual defects are one of the important indicator for the investigation of tumor progression in glioblastoma patients 13. Therefore, due to the coupling and interdependence characteristic of DR complications, the early diagnosis and detection of retinopathy in the diabetic individuals is imperative for the prevention of vision loss in patients.

The present review work started with a brief note on DR and its molecular mechanism with several pathological pathways. It was followed by studying the traditional and advanced treatment methods, chemical and natural drugs, and other novel therapies for treatment of DR. Subsequently, the imaging systems play a significant role in the early diagnosis and treatment. Therefore, we also compared various optical imaging modalities with their limitations. Optical coherence tomography (OCT) is one of the preferred ocular imaging systems, is also discussed. We discussed the advantages of the above hybrid imaging system in current ocular research. The final section briefly overview the importance of nanotechnology-based carriers that can be used in DR for effective nanotherapeutic ocular drug delivery applications.

## Diabetic Retinopathy (DR)

**Figure 1:**
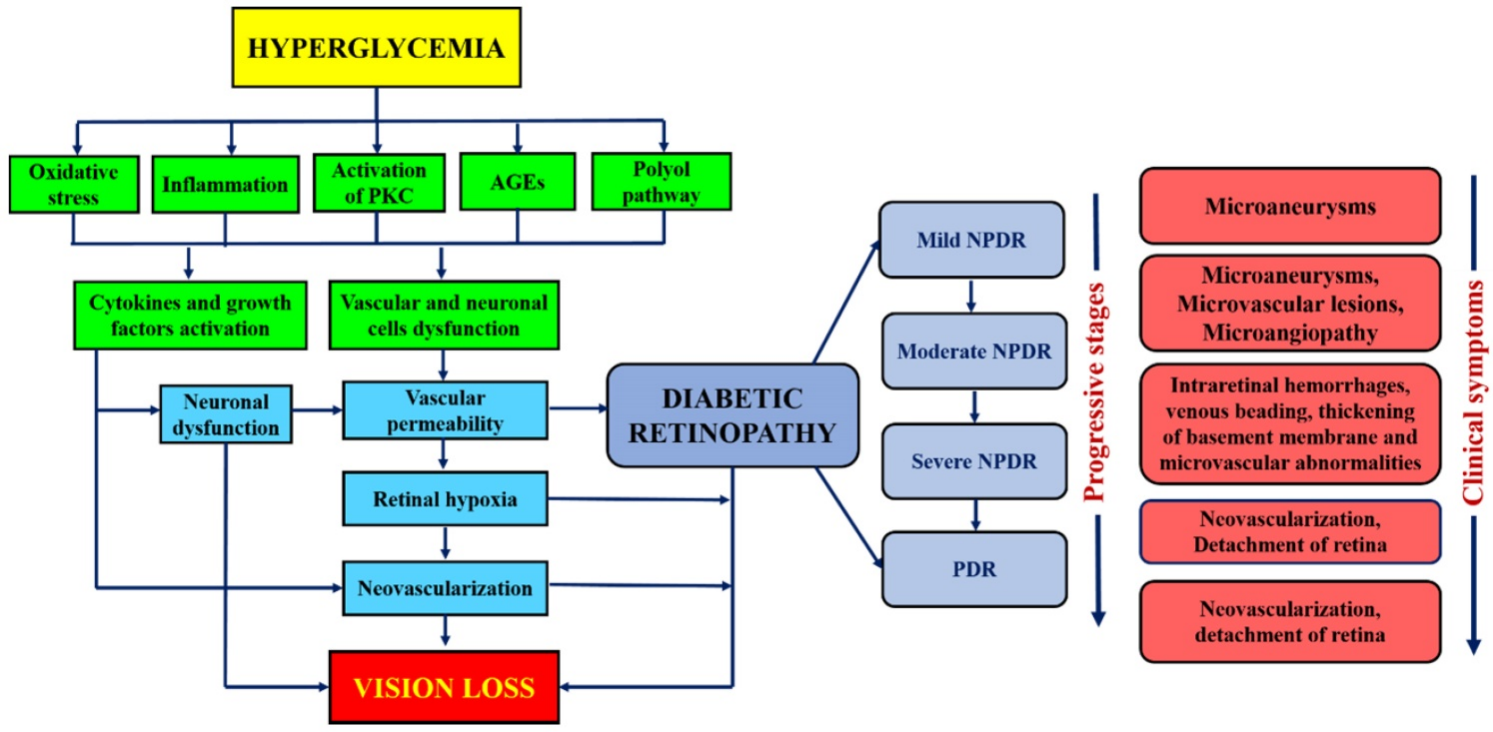
Flow diagram showing the major physiological factors involved in the pathogenesis and clinical symptoms at different stages of DR.

DR has been broadly classified into non-proliferative diabetic retinopathy (NPDR) and proliferative diabetic retinopathy (PDR) 14. NPDR has been ophthalmologically characterized by intraretinal hemorrhage, microaneurysm, venous, and intraretinal microvascular abnormalities 15,16. It can further lead to progressive stages such as mild, moderate, or severe NPDR, depending on the extent of the above-mentioned abnormalities. Besides, the progressive retinal ischemia and hemorrhage may lead to proliferative diabetic retinopathy (PDR). It is characterized by vitreous/pre-retinal hemorrhage and neovascularization. It also causes diabetes macular edema (DME) that leads to the leakage of blood and fluid into the retina due to the inflammation of the central retinal region. Another type of DR is the crystalline retinopathy that is associated with different genetic factors, etiology and degenerative causes. Its management approaches, imaging methods and various treatment startegies are earlier reported 17. Besides the molecular level, the natural mechanism of the DR progression in human and animal models is the regulation of blood glucose levels that tends to the hyperglycemia (elevated blood glucose level). The mechanism of hyperglycemia is elaborated in the flowchart shown in Figure 1. In both human and animal models, the chronic hyperglycemic condition does not cause any pathological changes in its initial 5-6 weeks of existence. The cell proliferation and swelling of retinal layers, however, can be observed. Unfortunately, the retinal lesions are shown to be persisted even after the glucose level became normal and thus led to the progression of DR 18. Therefore, it is essential to point out the role of biological phenomena that promote hyperglycemia. Besides, mitochondria, which is an important cellular organelle, that controls the energy metabolism (glucose). Its dysfunction also causes various disorders in eye, central nervous system, heart, etc. 19. Also, the trials conducted by three different agencies demonstrated that glycemic controls play a significant role in the prevention or delay of the onset of diabetic retinopathy 20-22. Beta cells of the pancreatic islets of Langerhans secrete insulin. This hormone is responsible for regulating the glucose level in the blood. The beta-cell dysfunction is due to several factors such as autoimmunity and inflammation, islet amyloid, lipotoxicity, adipokines, insulin resistance. The beta-cell dysfunction in type I diabetes is due to the autoimmunity of the T cells. The T cells attack beta cells causing approximately 70-90% loss. Whereas in type II, the dysfunction is complex and varies from asymptotic to symptoms of hyperglycemia. Apparently, both types substantially overlap and difficult to differentiate. Henceforth, the assessment of beta-cell mass and function is vital to understand the mechanism of the pathogenesis of diabetes and all its clinical complications that includes DR. Direct evaluation methods are thus not available. There are however various simple methods, and development of markers for the proper evaluation. These methods are important for understanding the pathogenic mechanism involved in the disease progression in animal models and human 23. Figure 1 summarizes the continuous flow diagram of all key factors involved in the pathogenesis of diabetic retinopathy (DR) and associated clinical complications.

### Molecular mechanism of DR

**Figure 2:**
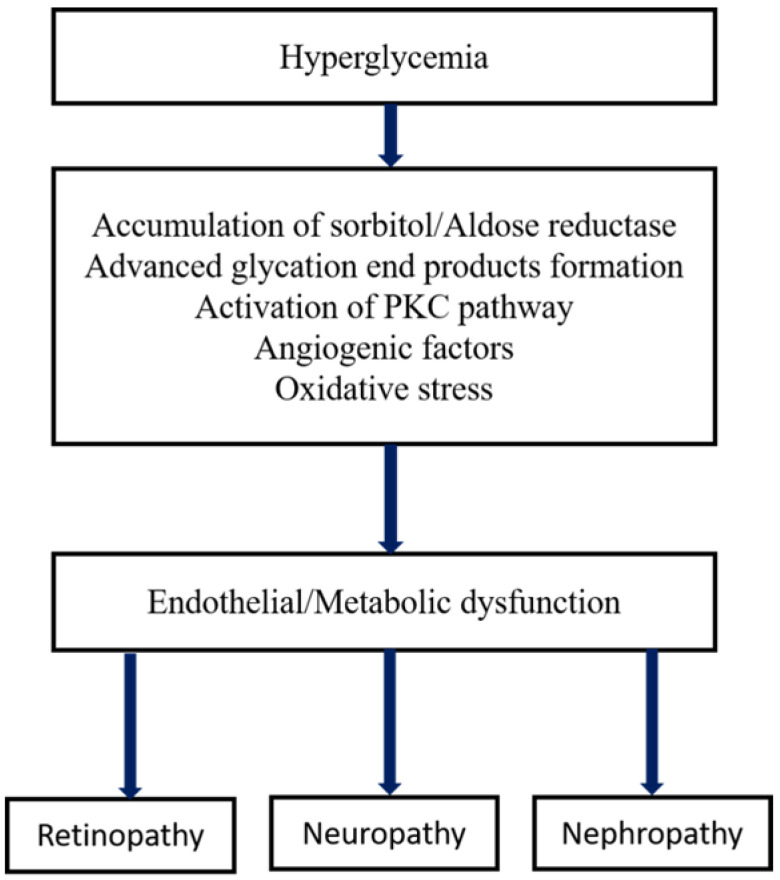
Metabolic pathways of diabetic microvascular complications 162.

In terms of its etiology and pathology, DR has enormous similarities with other neovascular diseases having chronic inflammation such as infiltration of immune cells, macular edema, vascular permeability, tissue destruction, and abnormalities in neovascularization. The primary risk factor for the development of DR is diabetes, in which Type I diabetes is more severe than type II that leads to vision loss. The other high-risk factors of DR are smoking, race, hyperlipidemia or dyslipidemia (excess fatty deposits in the blood), hyperglycemia, and hypertension (high blood pressure), etc. 24,25. The major pathological factors of DR include inflammation 26,27, up-regulated intercellular adhesion molecule-I (ICAM-1), enhanced leukostasis inside the retinal vessels 28, elevated vascular endothelial growth factor (VEGF) agents promotes reduced tight-junction proteins which leads to breaking of BRB 29, increased nitrous oxide synthase (NOS) 30 and inorganic phosphate's imbalanced metabolism 31. Several interconnected biochemical pathways play a significant role in the development of DR, such as increased hexosamine biosynthesis pathway (HBP), polyol pathways, the formation of advanced glycation end products (AGE), hemodynamic variations, and activated Protein kinase C (PKC) 32-34. Microvascular endothelial dysfunction is due to the RhoA/mDia-1 and RhoA/ROCK1 pathways in which RhoA act as GTPase. Activation of these pathways leads to initiated various pathological agents such as VEGF, insulin like growth factor (IGF), renin-angiotensin-aldosterone system (RAAS), occlusion of the capillary 28, increased ER and oxidative stress. Further, it was followed by de-regulation and autophagy 35, RPE dysfunction, epigenetic variations such as disruption in gene transcription, methylation of DNA and regulated non-coded RNAs 36,37,37,38. Interestingly, the depletion of transforming growth factor-β (TGF-β) signaling results in changes in the structure and function of the retina that mimics the characteristics of DR. Inhibition of gene functions like BMP2 (bone morphogenic protein 2) and Toll-like receptor 2 (TLR2) is also implicated in DR 39. The formation of plasma membrane pores activates the P2X7 receptor of the ligand gated ion channels leads to the development of DR through induced inflammation 40. Recent studies found that the high glucose (HG) and thioredoxin interacting proteins (TXNIP) were up-regulated in the retinal cells. In general, TXNIP overexpression causes severe pathological complications such as mitochondrial dysfunction, ROS stress, inflammation and premature cell necrosis due to the suppression of antioxidant property and thiol reducing ability of thioredoxin 41. Furthermore, hyperglycemia-mediated vascular dysfunction and the following tissue breakdown occurs through four main routes. 1) Elevated polyol pathway flux, i.e. increase in NADH^+^/NAD ratio via sorbitol pathway leads to the decrease in cytosolic NADPH and other cellular functions which leads to the cytosolic redox imbalance. 2) Increased AGE formations and glycosylation of proteins promotes alteration in the gene expression and induces the inflammatory cytokines. 3) Development of VEGF and other multiple growth factors due to the activation of PKC through diacylglycerol (DAG) and AGEs formation with intermediate ROS generation. Variation in the gene expression and protein function is attributed to the increased hexosamine flux occurs through conversion of fructose 6 phosphate to uridine diphosphate *N*- acetyl glucosamine. However, these major four metabolic pathways shown in Figure 2 are interlinked through the production of superoxide and elevated reactive oxygen species (ROS) generation which forms the platform for developing potential treatment strategy 42,43.

In clinical development, the success rate of several new promising drug candidates in human clinical trials remains lower. Such decline is due to the entry of a large number of drug interactions with less validated targets, lack of efficacy in intermediate stages of clinical studies. These intricacies can be improved by having stringent criteria during the non-clinical stages of the development of drugs and diagnostic tools. A prominent success in clinical trials would be achieved only through the animal models, which assists in the evaluation of target validation and efficacy of the drug candidates. Direct evaluation/testing of a newly developed compound for a specific human disease through human models will be a challenge to the human race. Therefore the selection of lower species from the animal hierarchy (non-human animal models), preferably mice, rats, rabbits, etc. would be scientifically valid complying with the animal ethical standards.

## Treatment

Several biomedical devices have been used for the detection, diagnosis, and treatment. Albeit the prevalence of advanced treatment techniques, the DR patients are having combined treatment options such as systemic monitoring of blood pressure and glucose level, laser-based photocoagulation, surgery, steroidal, and anti-VEGF intravitreal injections 44. Confocal laser scanning microscope, fluorescence angiography, ultrasound, and optical coherence tomography (OCT) are a few examples of biomedical imaging systems used for diagnosis. The imbalanced glycemic control can lead to the progression of clinical signs for DR and causes vision-impairing complications irrespective of intensive hypoglycemic treatment 45. Panretinal photocoagulation (PRP) is the common treatment method used to prevent progressive visual impairment in DR patients with high-risk characteristics for PDR. Denaturation of tissue proteins that causes the coagulation or the cell necrosis is thermally burned by argon laser to subdues the PDR symptoms. However, PRP is a relatively harmful treatment technique where it sacrifices the outer retinal layers such as retinal pigment epithelium (RPE), inner plexiform layer (IPL), and nerve fiber layer (NFL) in order to save the central vision 46. Consequently, focal photocoagulation slows down the frequency of persistent DME by blocking the fluid leakages in respective patients 47. Also, the laser photocoagulation, despite having the preventive potential for the progressive complications of DR, it addresses the pathological effects. Intravitreal injection, an invasive treatment method, involves the infusion of therapeutic agents such as anti-VEGF and steroids. Intravitreal administration of triamcinolone acetate (IVTA) gives the short-term benefits in comparison to the focal photocoagulation procedure in DME patients 47. Intravitreal administration of anti-VEGF agents such as pegaptanib, ranibizumab, bevacizumab, and aflibercept have a positive impact on vision by annihilating the age-related macular degeneration (AMD) caused due to choroidal neovascularization 48. These drugs exhibit significant efficacy by suppressing DME and restore the vision loss in the case of diabetic retinopathy (DR) 49-51. The chronic and relapsing characteristic of DME requires multiple successive intravitreal injections round the year. Also, the inhibition of VEGF agents activates the pathologic angiogenesis simultaneously, which are the trophological factors for the neuronal and endothelial cells 52,53. Henceforth, a non-invasive therapeutic tool has to be developed for the treatment of DR 54. Alongside, in order to protect ourselves from DR and minimize its potential complications, bio-imaging based theranostic approaches have to be developed with appropriate animal models before the clinical trials. Therefore we will be discussing the non-invasive imaging techniques in the later section.

### Treatment planning

The ophthalmologists and engineers worldwide are working towards achieving the theranostic objectives in order to overcome the current treatment limitations. The primary goal is the rehabilitation of the functional integrity of neovascular and BRB units 55. Various types of cells and their interactions between neurons, glial cells, and secretory mediators are involved in the neovascular units. When DR progresses with time, alteration of cellular interactions takes place with inflation in inflammatory cytokines and VEGF growth factors. Accordingly, an evaluation of the specific functions of various types of cells and mediators in DR promotes the development of pathogenesis mediated therapeutic procedures in DME. DR also causes retinal neovascularization or angiogenesis having characteristic changes that lead to the progression of new vessels along with the components of the vitreous cavity 56. In order to treat and avoid complications such as vitreous hemorrhage (VH), tractional retinal detachment (TRD), etc. due to retinal neovascularization, anti-VEGF agents can be used by targeting the synergy among the endothelial cells and vitreous proteins in specific 57.

## Treatment methods of DR

Various treatment methods of DR include laser photocoagulation (LP), injections of steroids, anti-VEGF agents through the intravenous route, and intraretinal surgery. The treatment options for DR are limited after the visible threats are observed in patients. Table 1 represented few conventional and new laser based treatment approaches with benefits and their adverse effects. LP is a surgical technique that suppresses the progression of DR, and vitrectomy helps in the prevention of vision loss from the advanced stages of DR. Vitrectomy is a conventional surgical procedure carried out for vitreous haemmorhage in retinopathic patients. Some of the complexities are vitreous removal, peeling of membrane, cataract, recurrent hemorrhage and neovascular glaucoma 58. This minimal invasive surgical technique got faded due to the advancements in intravitreal therapies using anti-VEGF agents. The main objective of LP is to reduce vascular leakage using the focal and grid laser burns in the BRB region. Macular focal laser photocoagulation is attributed to the DME, whereas the grid-based laser photocoagulation leads to the high-risk PDR 59-62. However, both these methods possess the disadvantage of additional vision loss. Also, a study reported that the focal/grid laser application to the central retina diminishes the prevailing vision rate by 50% in DME individuals 61,63.

In modern era, the dominancy of the intravitreous anti-VEGF injections demonstrated the therapeutic advancements in DR 64-66. Recent experiments suggested that the anti-VEGF agents include bevacizumab, aflibercept, and ranibizumab are found to be effective in the treatment of DR in terms of vision improvement 67,68. The anti-VEGF therapy is effective for retinal neovascularization with PDR, where it transforms the severe condition to the less developed state, and it is a viable alternative to PRP treatment 69. Also, the eyes treated with ant-VEGF agents shows better visual acuity in comparison to the PRP 70. The evidence for the treatment outcomes of the anti-VEGF therapy against neovascular AMD was reported in extent 71. The other advantages of anti-VEGF therapy are decreased rate of onset DME, less vision field loss, and reduced vitrectomies over time. Table 2 listed some of the essential anti-VEGF and anti-inflammatory drugs with advantages and their adverse effects. Diabetic Retinopathy Clinical Research Network (DRCR.net) evaluated the combinatorial impact of Laser-Ranibizumab-Triamcinolone in Diabetic Macular Edema (DME) and shown 50% positive outcomes in comparison to the alone focal/grid laser treatment. Both laser and anti-VEGF intravitreal injections, apart from its significant effects, had several adverse effects retinal damage, scar formation 72, endophthalmitis 73, intraocular inflammation 74, increase in intraocular pressure (IOP) 75. The vitreoretinal steroidal injection causes cataract development and enhanced intraocular pressure 76. The adverse side effects of these agents are caused both by the drug itself and its intravitreal route of administration and other local side effects are macular hole, uveitis etc. 77. All the treatment procedures are available only to the advanced stages of DR and were performed to prevent future vision loss; however, the retinal damage done is unavoidable 78.

### Natural compounds in treatment of DR

Several research works reported that the natural compounds always show promising effects against DR**.** Oral administration of curcumin suppresses the expression of VEGF in the retina of diabetic rats 79. Few herbal compounds play a significant role in diabetes, having hypoglycemic effect and prevents the secondary complications of DR owing to their high antioxidant properties. Some of the herbal drugs are Turmeric *(Curcuma Longa),* Fenugreek (*Trigonella foenum-graecum*), Tulsi (*Ocimum sanctum*)**,** Guduchi *Tinospora cordifolia* etc. Besides, there are several natural plants having hypoglycemic properties are shown in Table 3. The major advantage is that so far no adverse effects from the plants has been reported which is a safe naturally derived compounds for the management of diabetes. These natural compounds are not clinically approved hence there is no significant clinical evidence in the treatment of DR so far.

### Other treatment strategies for DR

There are several inhibitors such as fibrates, statins, thiazolidinediones, PKC β, ALR2 etc. are used in the treatment of DR and other complications. Fenofibrate Intervention and Event lowering in Diabetes (FIELD) study reported that the long-term treatment of fenofibrate reduces the progressive stages of DR and avoids the necessity for laser treatment through. Early treatment diabetic retinopathy study (ETDRS) using candesartan controls the progressive stages of DR in type I and II diabetes 80. Statins are profound to be effective in controlling the promotion of retinal inflammatory factors, and cytokines such as VEGF, ICAM's in diabetic eyes. Animal studies reported that both Lovastatin and Simvastatin were involved in the inhibition of pro-inflammatory transcription factor NF-κB and also inhibits the expression of VEGF and VEGF induced intercellular adhesion molecule. Thiazolidinediones are insulin-sensitized peroxisome proliferator-activated receptor PPAR-γ agonists, commonly known as Glitazones. Its anti-inflammatory action decreases the fatty acids in plasma that have a significant effect on the vasculature, which makes it suitable for the treatment of DR. Few examples of thiazolidinediones available in India are rosiglitazone, pioglitazone, etc. Several synthetic and natural orally active drugs are used for the inhibition of PKC*β* regulators for controlling the microvascular complications in diabetes. Midostaurin is naturally available alkaloid staurosporine, which inhibits the kinases of PKC. The animal studies reported its inhibitory action against neovascularization. However, shown hepatotoxicity due to the non-specificity of the drug 81. Similarly, Ruboxistaurin is an orally active protein kinase β inhibitor used for the treatment of microvascular complications of DR. It also had a mild effect on DME, and the outcome of the clinical trials (random) shown the functional visual improvement and 50% of DR can be prevented 82,83. It has to be noted that the above conclusions are derived from clinical and animal experimental studies, hence these inhibitors are not clinically approved for treatment. Apart from this, there are several advanced methods such as stem cell therapy, gene therapy, and other regulating factors for controlling the ocular complications are still in the research levels which are being reported by various research works 84-86.

An integrated approach is necessary for the reduction of mortality rate due to DR^87^. Development of effective treatment strategy is essential for early prevention of visual impairment and show constant improvement in the vision. Henceforth, the clinical researchers are putting persistent efforts in pursuit of answers for the undisclosed mystery involved in DR treatment and its complications. A suitable example is that 40-50% of cases with DME do not respond to anti-VEGF therapy. This kind of therapeutic gap necessitates the clinical engineers to develop advanced state of the art theranostic systems based on non-invasive, nondestructive treatment methods, which will be the future platform for clinicians.

## Imaging Biomarkers

Eye is one of the appropriate candidates for being the optical window for the investigation of ocular abnormalities in small animal models and its associated neurodegenerative complications and tumor as well. These studies are to be carried out through non-invasive imaging tools without the need for any biopsy. These imaging techniques become the privilege for either human or animal models, which is ethically safe and can be studied until the natural decease of the animals.

Over the decade, the development of various advanced imaging modalities made it possible for the routine imaging of human retina *in vivo* at subcellular resolution 88. Ophthalmic imaging is still been used in small animals for translational research. Various imaging modalities such as optical imaging, radionuclide imaging, magnetic resonance imaging, computed tomography, ultrasonography, etc. are used in ophthalmology and other applications 89. The non-invasive imaging and optical modalities such as bioluminescence, reflectance, magnetic resonance spectroscopy imaging (MRSI), positron emission tomography (PET), and contrast-enhanced perfusion MRI have their intrinsic limitations for the investigation of ocular and tumor-related neovascularization 90-95. The human eye, by nature, promotes the sub-cellular mediated resolution and non-invasive *in vivo* imaging of the retina and its vascularization in healthy and diseased states (retinal tumor, DR, uveitis, etc.). It has its in-built natural vision with imaging optics, cornea, and the crystalline lens 88,96,97. *In vivo* imaging of mouse retina using computed tomography based adaptive optics (AO) enhanced OCT/SLO system has also been reported recently 98-101. Imaging of the retinal layers using the advanced techniques is challenging due to the non-availability of the sophisticated non-invasive system where the retinal size animal models is ten-fold smaller than that of humans. Also, there are very limited in vivo ocular non-invasive imaging methods available for retinopathy with in depth resolution 102-107. Recently, an early detection of DR using a non-mydriatic, full field electroretinopathy, easy to operate devise was reported. This devise is used as an adjunctive tool for the screening of DR 108. Similarly, there are several computer aided diagnosis of DR has been reported 109,110. OCT based imaging and diagnosis of DR in animal models are limited.

**Figure 3:**
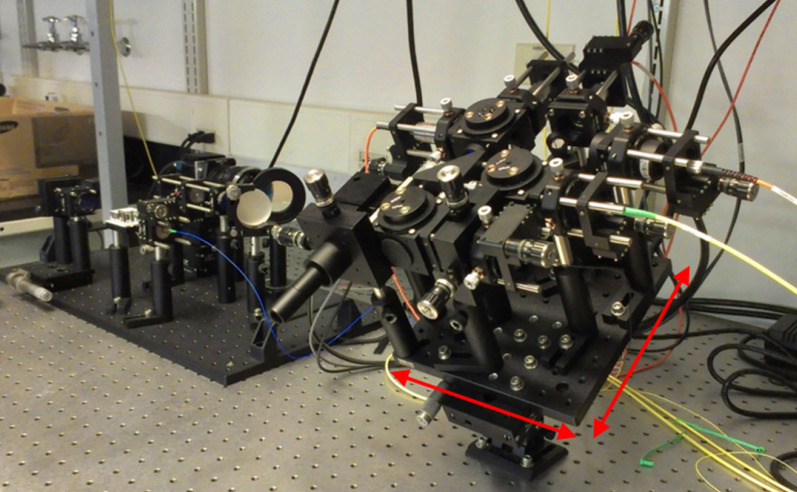
Digital photograph of combined OCT/SLO imaging system.

**Figure 4:**
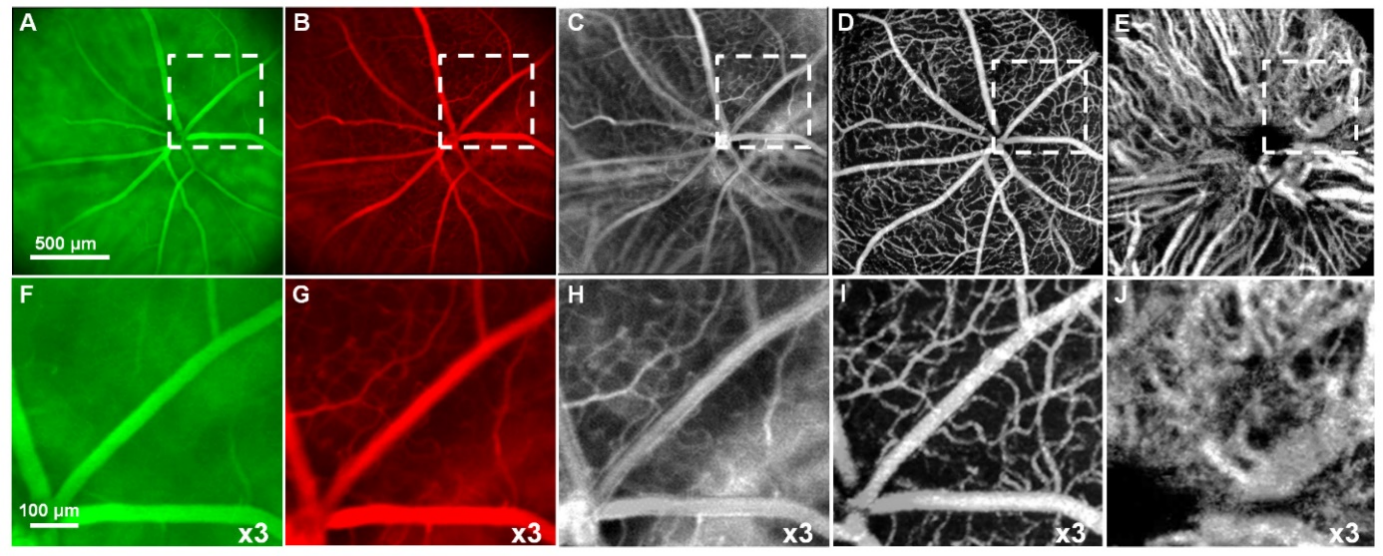
Simultaneous SLO and OCT imaging of a nude mouse with two fluorescence channels injected with fluorescein and nanoparticles tagged with rhodamine-X: (A) Image of ”green” (“GFP”) fluorescence channel; (B) Image of ”red” fluorescence channel; (C) Image of the ratio between (B) and (A); D Image of the retinal vasculature extracted from OCT (pv-OCT); (E) Image of the choroidal vasculature obtained from OCT. (F-J) x3 magnified images of the white dashed area from Figure 4A-E respectively showing enhanced visualization of chorioretinal vasculature. (*Adapted from* Goswami et al. 2016 163).

Optical coherence tomography (OCT) is a retinal imaging tool that uses coherent infrared laser by analyzing the reflectance properties of a sample with in-depth resolution. In short, OCT rapidly procreated the volumetric representation of the intrinsic retinal layers and vasculature in three-dimensional resolutions of few micrometers. Some of the uncommon features of AMD in eyes such as retinal tabulation, cystoid degeneration and choroidal cavern were also reported through OCT 111. In contrast, the scanning laser ophthalmoscopy (SLO) rendered the confocal reflectance and fluorescence images 112-114. The systematic digital photograph of such an hybrid OCT/SLO imaging system of our research group was represented in Figure 3. The research work has reported the potential of this combined OCT/SLO system. Figure 4(A-J) shows the angiography of retina and choroid through green fluorescing fluorescein and micellar nanoparticle tagged with red fluorescing rhodamine-X. Comprehensively, a combined OCT/SLO system comprises AO-SLO for imaging physiological events of retinal components such as blood flow, microglia cells, capillaries, GFP expression of cone photoreceptor cells and its three-dimensional localization by combining with the widefield OCT/pv-OCT and SLO records. Similarly, Optical coherence tomography angiography (OCTA) is an innovative utilization of OCT for the visualization of retinal layers without use of dyes 115. It has several features such as increased acquisition speed and image information over the dye based angiographic system 116.

**Figure 5:**
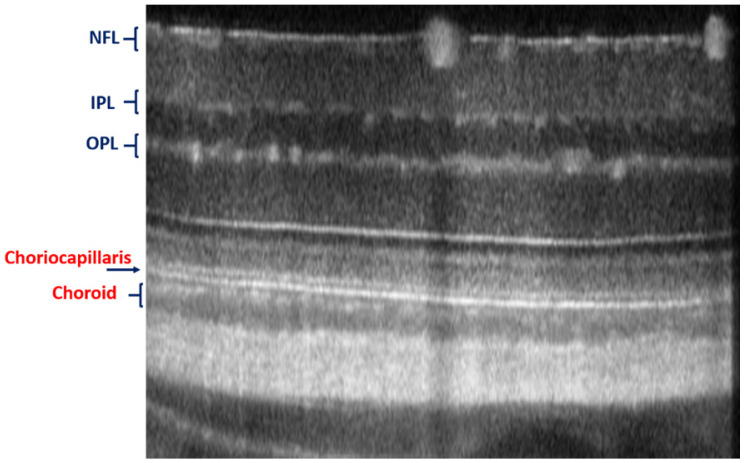
Imaging of choriocapillaris through OCT/SLO system.

**Figure 6:**
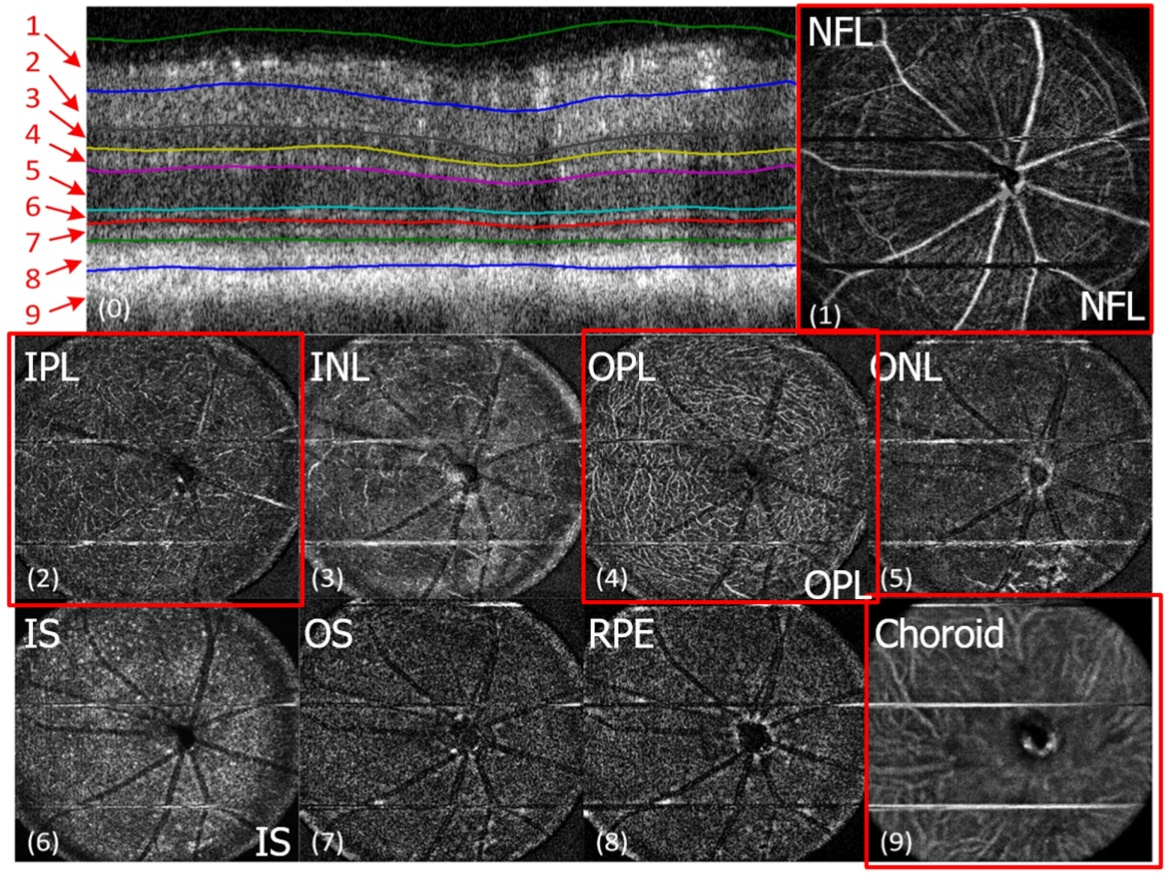
*In vivo* imaging of microvasculature layers and depth localization through pv-OCT/SLO system. Micrographs showing pv-OCT/SLO imaging showing images visualized at different layers such as nerve fiber layer (NFL), inner plexiform layer (IPL), inner nuclear layer (INL), outer plexiform layer (OPL), outer nuclear layer (ONL), inner segment (IS), outer segment (OS), retinal pigment epithelium (RPE), and choroid, respectively. (*Adapted from* Goswami et al. 2016 163).

The advantages of these two systems have been complied with the natural optical systems like in mouse eye, amalgamating their capabilities, and promotes *in vivo* mouse studies for probing the structural and functional information of the retinal unit 117,118. The utilization of such fusion adaptive optics (AO) for imaging is an ongoing revolution in bio-imaging research in which structural and functional visualization of cells can be done *in vivo* through the genetically encoded optical probes^119^. AO-SLO is one of the most appropriate imaging biomarkers used in recent times designed exquisitely for the fluorescence and reflectance imaging of the retina of the mouse 101. The utility of this imaging system could be demonstrated for the imaging of retinal capillaries, microglia, retinal blood flow, and cone photoreceptor cells, which collectively exhibit green fluorescence protein (GFP). This further illustrates the localization and visualization of the morphological structures and cells in 3D by combining the AO-SLO data with wide field OCT/phase variance OCT and SLO data. The studies performed on such a combined imaging system reported the extensive differentiation and visualization of microvascular retinal layers, choriocapillaris represented in Figure 5 and Figure 6, respectively.

In general, the custom OCT system comprises of a superluminescent diode as light source, a spectrometer with a diffraction grating, an imaging objective, CMOS camera with an operational range up to 100,000 A-scan/s as its detector. It also consists of a sample arm with fiber collimator lens, galvanometric scanners and achromatic lens, reference arm consist of fiber collimator lens, neutral density filter, achromatic lens, a static silver coated mirror and dispersion-compensating block. Theoretically, the OCT system has the axial and lateral resolution (~2 µm and ~5 µm) having image acquisition of a visual angle over 41 degrees (~1.4 mm) 120. Similarly, a custom-made SLO multichannel system is used for the acquisition of reflectance and fluorescence images. It possesses an essential feature of the spectrum analyzer for the fingerprinting of autofluorescence and fluorescent cells through spectral emission. The images were combined with the OCT retinal vasculature data, followed by the localization of fluorescent cells and retinal microvasculature events in higher resolution in AO-SLO imaging 121. A general method was developed using the pv-OCT and AO-SLO system for the localization and characterizing the retinal microvasculature. It was mainly used for localizing the fluorescent microglia cells of different retinal layers. Several studies on the combined pv-OCT/SLO imaging system with fluorescent angiography, retinal neovascularization in retinopathy and tumor *in vivo* models has been reported recently 122.

The above study also have reported the fluorescence images and mapping of the retina in which the AO-SLO images are localized within the distinct axial focal planes and patterns of knocked GFP expressed cells. The microglia-mediated inflammation has been reported in the number of disease etiologies indicated by its migration and morphological changes 123. These morphological variations of the microglia from highly branched to amoeboid shape along the axis through the retinal layers can also be visualized by using the AO-SLO system. Apart from studying the microglia response to the injury, this gives significant *in vivo* proof for the role of microglia that provides insightful scientific evidences required for treatment of DR and other ocular complications.

## Nanotechnology in Ocular Drug Delivery

Ocular drug delivery remains an formidable challenge to researchers despite several efforts 124,125. The development of an effective ocular drug delivery system becomes an important objective for pharmaceutical engineers and scientists. Most of the frontal ocular diseases are treated through topical and sub-conjunctival administration of drugs in the form of solutions, ointment, and suspensions. Whereas, intravitreal injection of drugs is the predominant route of administration for the posterior ocular diseases. Administration of drugs into the intravitreal region are still facing poor ocular bioavailability owing to the pathophysiological and anatomical barriers in the eye. Recent studies urged the importance of nanotechnology-based drug delivery carriers such as nano-emulsions, nano-suspensions, dendrimers, liposomes, niosomes, cyclodextrin, etc. for the effective treatment and therapy for ocular complications 126-129. The current promising drug delivery systems are nanomicelles, nanodevices, soft contact lens, bioadhesive systems, microfabricated systems, implants, cell encapsulation devices etc. 130-133. Nanotechnology offers targeted drug delivery and sustained release of the therapeutic drug at the site 134. The objective of such targeted delivery and controlled release is the effective management of pharmacokinetics, pharmacodynamics, immunogenicity, and non-specific toxicity.

### Nanomedicine

Nanomedicine plays a significant role in diagnosis and novel approaches for therapeutic ocular drug delivery 135,136. The use of nanotechnology-based drug delivery systems such as nanomicelles, solid lipid nanoparticles (nanospheres and nanocapsules), nano-suspensions, and drug loaded nanocarrier (Nanodrug) gives better solution to solubility and bioavailability of various poorly soluble therapeutic drugs especially in the posterior ocular diseases 137. These drugs can also be targeted to allow the site-specific delivery and minimal side effects to the nearby cells 138. Table 4 represented the suitable ocular nano drug delivery formulations like liquid, semi-solid or solid forms, route of drug administration, drug loading dosage and dosage intervals, release and retention time, drug concentration in vitreous for various retinal diseases. The particles, gels and implants are different drug formulations for intravitreal injections for posterior ocular diseases. Nanodized drug delivery systems distribute the drug at the target cells. The injected nanodrug carriers should be toxic free, with physiological pH, should not induce aggregation in the vitreous fluids. Clinically used therapeutic compounds are simple lquid form (Ranibizumab or bevacizumab) or implants. The degradable (Ozurdex) and the non-degradable polymeric implants (Iluvein, Retisert) are eliminated from the anterior or posterior route. The presence of enzymes such as metalloproteinases, heparinase etc. could contribute in the polymeric degradation however sparse information is available on the elimination route of non-degradable implants 139-142. Gels are used in drug delivery owing to their sustained drug release into the vitreous region. Thermoresponsive hydro/nanogels like PLGA-PEG-PLGA are attractive with dual characteristics of aqueous form during injection and gel formation inside the vitreous 143. Sustained drug release can be extended by incorporating into particles (polymeric miscelles) within gels 144,145. Moreover, the utilization of hyluranic acid as gelling material is an effective method for drug delivery, but cationic particles should not be chosen as it forms aggregation in the vitreous humor 143.

Potential tissue barriers for ocular drug delivery are cornea, sclera and conjunctiva in frontal site. Whereas in the back of the eye are vitreous, inner limiting membrane (ILM), retinal pigment epithelium (RPE) and choroid. The frontal barriers consist of tight junctions in which small and large molecules upto 5kDa can permeate 146,147. On the contrary, small nanoparticles, lipophilic compounds, drugs, proteins can diffuse easily into the posterior barriers 148-151. Similarly, the vitreous-retinal barrier plays major role in nanoparticles drug delivery to retinal region. It depends upon various properties of carriers such as particle charge, surface structure, and hydrophobicity. Size of the injected particles affects the diffusion into retina and vitreous. The vitreous having pore size of 550 nm allows diffusion of large particles than ILM (having smaller mesh size of 10-100 nm in human). Drug delivery nanocarriers are essential for free drugs which is not able to reach its target site. Such nanocarriers are capable of diffuse and penetrate into both vitreous and ILM owing to their tunable properties. It is important that the design of nanostructures with anionic and neutral surface charge is used. Also, these surface charged nanoparticles of mean size 132-350 nm find it difficult for retinal penetration 152,153. This could be due to non-uniform monodispersion of nanoparticles, i.e, only the smaller nanoparticles are able to penetrate into the retina. Few research works reported that most consistent retinal penetration is visible in soluble molecules such as PCL-PEG miscelles as ideal drug delivery carriers. These have effective intracellular properties; however the propertics may vary with different target retinal cell types 142,154. The other polymeric nanomaterials also find enormous application in the treatment of DR and its several complications are poly (lactic acid) (PLA), chitosan, gelatin, poly (methyl methacrylate) (PMMA), poly (2-hydroxyethyl methacrylate) (pHEMA), and polyethyleneimine (PEI)-Chitosan.

The application of nanomaterials is emerging in ocular research especially in the treatment and therapies of DR. Nanoparticles such as silver, gold, titanium dioxide have their intrinsic therapeutic properties and potentials 155. Early literature also shows that these metal based nanoparticles are being used in the treatment of DR 156-158. Retinal neovascularization is the most critical factor in DR pathogenesis. The anti-angiogenic effects of silicate nanoparticles induced by VEGF on retinal neovascularization were tested 159. It has been found that no toxic effects on retinal tissues on administration, and reduces retinal neovascularization. Hence, these nanoparticles are suitable for the treatment of VEGF induced neovascularization.

**Figure 7:**
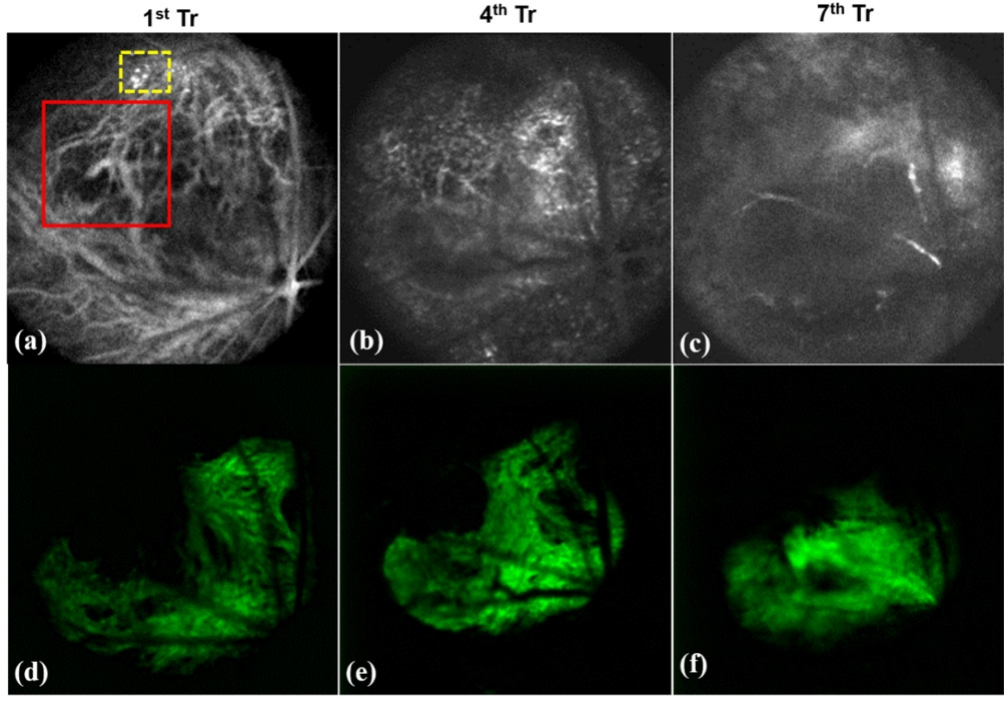
Visualization of porphyrin nanoparticles localized vasculature of *in vivo* mice showing (a-c) OCT images of the neovasculature (new tiny blood vessels) localized by porphyrin nanoparticles (as in yellow box) treated on 1^st^, 4^th^ and 7^th^ day and (d-e) represented its respective green filter images. (Red box in Figure 7a showing the normal blood vessels which is larger than the newly formed vasculature).

Fluoresecent based nanomaterials for non-invasive *in vivo* imaging plays major role in early diagnosis and treatment planning. Some of the fluorescent materials are Rhodamine, porphyrin, fluorescein isothiocyanate (FITC), green fluorescence protein (GFP), etc. Our former research groups have carried out imaging based diagnosis of tumor neovascularization in retinal layers through OCT using fluorescent nanoparticles. Porphyrin nanoparticles were used for the real time tracking of neovasculature *in vivo*. Figure 7 shows the representative OCT images of the tumor development in retina of mice showing the encapsulation of porphyrin with the newly formed tiny blood vessels of the retina (shown in dotted yellow box of Figure 7a). In the meanwhile, the normal retinal blood vessels are larger in size (shown in red box of Figure 7a) and Figure 7d-e represented the respective green filter images. The combined approach of non-invasive imaging based early diagnosis, nanotherapy is essential in the modern medical era.

Henceforth, the future perspectives of nanotechnology found to have multiple applications in ophthalmology. It helps in the development of nanodevices and nanocarriers for the treatment of various ocular complications in specific diabetic retinopathy (DR) and its associated complications. Furthermore, nanotechnology promotes the development of a 3D contact lens incorporated with nanomaterial encapsulated drugs for efficient drug delivery and treatment. 3D printing technology simplifies the development of therapeutic devices using either polymeric or metallic forms. Also, it will be helpful for medical engineers for the treatment and therapy using advanced tools 160. This technology has enormous potential and possesses very high impact on the manufacturing of customized 3D printed ocular prototypes such as the retina, lens, cornea in the near future 161.

## Conclusion and future directions

Diabetic retinopathy (DR) is one of the vital ocular complications generally occurred among the middle-aged people worldwide. The current treatment methods of DR involve intraocular or intravitreal chemotherapeutic injections, laser therapy, which leads to several post-treatment risks. Subsequently, stem cell therapies are though effective, but a pre-emptive nano-theranostic tool has yet to be developed. Presently our group is working on the research project on the development of a combination of non-invasive early diagnostic imaging system with nano-drug delivery approaches which are quintessential for effective ocular repair. This strategy could be an appropriate alternative to conventional treatment. Herein, this review work comprehensively discussed the diabetic retinopathy, its mechanism, biomedical imaging modalities, drugs (both natural and chemical), present and future perspectives of DR treatment and nanomaterials based ocular drug delivery approaches. Apart from the review of the current approaches, this work necessitates the essentiality of innovative non-invasive nano-theranostic imaging system and nano-drug delivery approaches for DR to achieve effective outcomes in patients. We also recommended the utilization and importance of 3D printing technology in the ocular research. This leads to the development of non-invasive nano-therapeutic and biomedical implants essential for the future clinical diagnosis and treatment of various ocular complications.

## Figures and Tables

**Table 1:** Laser treatment methods of DR

Type	Drugs	DR treatment status	Benefits	Adverse effects
Conventional laser treatment	Focal/grid laser 61	Adjuvant treatment for DME	Reduce the risk of moderate visual loss, increases the chance of visual improvement, decreases the frequency of persistent macular edema (Early Treatment Diabetes Retinopathy Study (ETDRS))	Visual acuity loss; visual field loss
	PRP 164	Adjuvant treatment for PDR with high-risk complications	Reduce the rate of severe visual loss in PDR and inhibit the progression of retinopathy (Diabetic Retinopathy Study (DRS))	Visual acuity loss; b. Constriction of peripheral visual field
New laser approaches	PASCAL 165	Under clinical evaluation	Accurate control of the laser; less treatment time	-
	D-MPL 166	Under clinical evaluation	Collateral damage is minimal	-
	NAVILAS 167	Under clinical evaluation	High accuracy of laser spots	-

**Table 2:** Anti-VEGF and Anti-inflammatory drugs for Treatment of DR

Classification	Drugs	Benefits	Adverse effects
Anti-VEGF drugs	Ranimizumab (Lucentis) 168,169 (FDA approved)	a) Huge BCVA improvement and high reduction in CRT over laser treatment for DME. b) Non-inferior to PRP in the treatment for PDR for 2 years	Vitreous hemorrhage; increased intraocular pressure (IOP) inflammation 170.
	Pegaptanib (Macugen) 171 (FDA approved)	BCVA improvement, in DME patients	Increased IOP; conjunctival hemorrhage
	Aflibercept (EYELEA) 65 (FDA approved)	High reduction in CRT and better visual acuity	Increased IOP; vitreous hemorrhage inflammation 170.
	Bevacizumab (Avastin) 170 (FDA Approved)	BCVA improvement and reduction in CRT in DME patients	Tractional retinal detachment (TRD); vitreous hemorrhage; increased IOP; inflammation.
Non-specific Anti-angiogenic drugs	Squalamine 172	-	-
	Razuprotafib AKB-9778 173 (Phase II trial )	Reduction in CRT in combined use over the Ranibizumab alone therapy	Worsen diabetic retinal edema; reduction in visual acuity
	Nesvacumab (Phase II clinical trial)	No differentiation in combined and Alflibercept alone therapy	No new safety indications observed
	Faricimab (Phase III clinical trial)	BCVA improvement and CRT reduction over Ranimizumab in DME patients	No new safety indications observed
Intravitreal steroids	Triamcinolone 64 (FDA approved)	Huge improvement in Triamcinolone + laser group over the control in pseudophakic eyes	Cataract surgery; high intraocular pressure (IOP); vitreous hemorrhage.
	Ozurdex 174,175 (FDA approved)	BCVA improvement, reduction in CRT in DME patients	Cataract; increased IOP; vitreous hemorrhage.
	Iluvein 176 (Clinically approved implant)	BCVA improvement in DME patients	Cataract; increased IOP; vitreous hemorrhage.

BCVA: best corrected visual activity, FDA: Food and Drug Administration, U.S.A.

**Table 3:** Natural therapeutic compounds for treatment of DR

Plants(*)	Active part and compound	Study
*Allium cepa* 177; *Allium sativum* 178; *Catharanthu roseus and* *Coccinia indica* 179; *Cyamopsis tetragonolobus* 180; *Ficus bengalensis* 181; *Momordica charantia* 182-184; *Trigonella foenumgraecum* 185*^i^*	Bulb, Alkyldisulfides; Bulb, Alkyldifulfides; Leaf, Alkaloids; Seed and pod, Alkaloids; Stem bark, Alkaloids; Aerial, glycosides; Seeds, Trigonelline	Diabetic rabbits; healthy rabbits; healthy rats; diabetic animals; diabetic rodents; NIDDM patients; IDDM and NIDDM patients

*Clinically not approved.

**Table 4:** Intravitreal administration of various Nano drug delivery systems

Material	Formulation	Drug compound	Drug loading (µg/mg) or total dose (µg)	*In vitro* Release time (days) or release rate (µg/day)	Time duration of action (days)	Concentration in vitreous (µM)	Ref
Chitosan coated PLGA	Solid Implants	Methotrexate	400 µg/mg; 400 µg		33 days	0.1 - 1.0	186
PVA-Silicone laminate		Fluocinolone acetonide	590 µg	0.3 - 0.6 µg/d	2.5 - 3 years	0.2 - 0.4	187
PVA, ethylene vinyl acetate		Ganciclovir	4.5 mg	1.4 µg/h	150 - 240 days	7.5 - 29	188
Silicone, poly vinyl acetate		Fluocinolone acetonide	190 µg	0.2 µg/day	728 days	0.1 - 2	189
PLGA		Dexamethasone	700 µg	1 µg/h	90 days	0.6	190,191
PLGA-PEG-PLGA gel	Semi-solid	Dexamethasone	1 mg/mL	> 10 days	>9	3 - 23	192
PLGA nano/microparticles	Liquid form	Dexamethasone Bevacizumab	40 µg/mg; 200 µg 12.5 mg/mL of suspension; 62.5 µg		>20 >30	21 - 31 63	193 194

## Abbreviations

DR: diabetic retinopathy
BRB: blood retinal barrier
OCT: optical coherence tomography
NPDR: non-proliferative diabetic retinopathy
PDR: proliferative diabetic retinopathy
DME: diabetic macular edema
ICAM-1: intercellular adhesion molecule-I
VEGF: vascular endothelial growth factor (VEGF)
NOS: nitrous oxide synthase
HBP: hexosamine biosynthesis pathway
AGE: advanced glycation end products
PKC: protein kinase C
RhoA: rhodamine A
ROCK1: rho-associated coiled-coil-containing protein kinase 1
GTPase: guanine triphosphate hydrolase
IGF: insulin like growth factor
RAAS: renin-angiotensin-aldosterone system
ER: endoplasmic reticulum
RPE: retinal pigment epithelium
DNA: deoxyribonucleic acid
RNA: ribonucleic acid
TGF-β: transforming growth factor-β
BMP2: bone morphogenic protein 2
TLR2: Toll-like receptor 2
HG: high glucose
TXNIP: Thioredoxin interacting proteins
NAD: nicotinamide adenine dinucleotide
NADH^+^: nicotinamide adenine dinucleotide dehydrogenase
NADPH^+^: nicotinamide adenine dinucleotide phosphate dehydrogenase
DAG: diacylglycerol
ROS: reactive oxygen species
PRP: panretinal photocoagulation
ILM: inner limiting membrane
IPL: inner plexiform layer
and NFL: nerve fiber layer
IVTA: intravitreal administration of triamcinolone acetate
AMD: age-related macular degeneration
VH: vitreous hemorrhage
TRD: tractional retinal detachment
LP: laser photocoagulation
DRCR: Diabetic Retinopathy Clinical Research Network
ALR2: aldose reductase
FIELD: Fenofibrate Intervention and Event lowering in Diabetes
ETDRS: early treatment diabetic retinopathy study
PPAR-γ: peroxisome proliferator-activated receptor
MRSI: magnetic resonance spectroscopy imaging
PET: positron emission tomography
AO: adaptive optics
SLO: scanning laser ophthalmoscopy
OCTA: optical coherence tomography angiography
GFP: green fluorescence protein
CMOS: complementary metal oxide semiconductor
PLGA: poly(lactic-co-glycolic acid)
PEG: poly(ethylene glycol)
PLA: poly(lactic acid)
PMMA: poly(methyl methacrylate)
pHEMA: poly (2-hydroxyethyl methacrylate)
PEI: polyethyleneimine
FITC: fluorescein isothiocyanate
